# Guilt is effectively induced by a written auto-biographical essay but not reduced by experimental pain

**DOI:** 10.3389/fnbeh.2022.891831

**Published:** 2022-08-11

**Authors:** Selina Schär, Antonia Vehlen, Julia Ebneter, Nathalie Schicktanz, Dominique J. F. de Quervain, Lutz Wittmann, Lutz Götzmann, Martin grosse Holtforth, Sonja Protic, Alexander Wettstein, Niklaus Egloff, Konrad Streitberger, Kyrill I. M. Schwegler

**Affiliations:** ^1^University Hospital of Child and Adolescent Psychiatry and Psychotherapy, University of Bern, Bern, Switzerland; ^2^Abteilung für biologische und klinische Psychologie, University of Trier, Trier, Germany; ^3^Psychology Department, University of Bern, Bern, Switzerland; ^4^Division of Cognitive and Molecular Neuroscience, University of Basel, Basel, Switzerland; ^5^International Psychoanalytic University, Berlin, Germany; ^6^Institute of Philosophy, Psychoanalysis and Cultural Studies, Berlin, Germany; ^7^Psychosomatic Medicine, Department of Neurology, Inselspital, University Hospital, Bern, Switzerland; ^8^Institute of Criminological and Sociological Research, Belgrade, Serbia; ^9^Department of Research and Development, University of Teacher Education Bern, Bern, Switzerland; ^10^Department of Anesthesiology and Pain Medicine, Pain Center, Inselspital, Bern University Hospital, University of Bern, Bern, Switzerland

**Keywords:** chronic pain, trauma, stress, moral emotions, emotional memory, pain-proneness

## Abstract

**Introduction:**

The aim of the present study was (1) to validate the method of guilt-induction by means of a written auto-biographical essay and (2) to test whether experimental pain is apt to alleviate the mental burden of guilt, a concept receiving support from both empirical research and clinical observation.

**Methods:**

Three independent groups of healthy male participants were recruited. Group allocation was not randomized but within group pain/sham administration was counterbalanced over the two test-days. Groups were tested in the following consecutive order: Group A: guilt induction, heat-pain/sham, N = 59; Group B: guilt induction, cold-pressure-pain/sham, *N* = 43; Group C: emotionally neutral induction, heat-pain/sham, *N* = 39. Guilt was induced on both test-days in group A and B before pain/sham administration. Visual analog scale (VAS) guilt ratings immediately after pain/sham stimulation served as the primary outcome. In a control group C the identical heat-pain experiment was performed like in group A but a neutral emotional state was induced.

**Results:**

A consistently strong overall effect of guilt-induction (heat-pain: *p* < 0.001, *effect size r* = 0.71; CPT-pain *p* < 0.001, *r* = 0.67) was found when compared to the control-condition (*p* = 0.25, *r* = 0.08). As expected, heat- and cold-pressure-stimuli were highly painful in all groups (*p* < 0.0001, *r* = 0.89). However, previous research supporting the hypothesis that pain is apt to reduce guilt was not replicated.

**Conclusion:**

Although guilt-induction was highly effective on both test-days no impact of pain on behavioral guilt-ratings in healthy individuals could be identified. Guilt induction per se did not depend on the order of testing. The result questions previous experimental work on the impact of pain on moral emotions.

## Introduction

Moral emotions are of utmost importance for individual life and social interaction and thus a precondition for cultural achievements. However, guilt and shame can derail and become pathologic, like encountered in biographies with abuse, trauma and loss (Wilson et al., 2006) where they are frequently accompanied by chronic wide spread pain (Egle et al., 2016). This work investigates the properties of experimental guilt induction by means of an autobiographical essay and-alluding to the Freudian concept of moral masochism (Freud, 1998)-tests whether pain alleviates the mental burden of guilt in healthy subjects.

### Negative moral emotions: Guilt and shame

Perceiving guilt and shame enables moral judgement and behavior with respect to relevant social contexts. Since both emotions are also strongly self-reflective they have been termed “self-conscious” (Tangney et al., 2007). Engel (2008), referring to psychoanalytic theory points out that *immoral* guilt denotes a conscious perception, which is due to an actual moral transgression, whereas *amoral* guilt remains largely unconscious and is related to what has been termed “moral masochism”. In this regard it is important to state that the present study, as well as the empirical research cited here, obviously deals with *immoral*, i.e., conscious aspects of guilt. In stricto sensu meaning that in our actual context the term “moral masochism” can only be used with restrictions.

However, whereas conscious guilt is usually triggered by an overt transgression, shame is more frequently related to a situation, which is only perceived subjectively as morally reprehensible. Hence, excessive shame condemns the self and hinders extravert compensatory behavior. Quite contrarily, the consciously guilty subject is able to better distinguish between self and behavior when reparative action along with remorse and apology serve for rehabilitation (Lewis, 1971; Tangney et al., 2007). Importantly, traumatic biographical experiences, early life stress and drastic losses can elicit pathologic guilt and shame (Wilson et al., 2006; Hutson et al., 2015; Lopez-Castro et al., 2019; Shi et al., 2021).

### Pain chronicity and trauma

The transition from acute to chronic pain is still not well understood. Nonetheless, it is clear that chronic pain links to long-term stress exposure (Vachon-Presseau et al., 2013; Egloff et al., 2014; Egle et al., 2016) and chronicity implies a shift of neural activity from neo-cortical to older meso-limbic areas linking to the stress-axis and to emotional memory function (Hashmi et al., 2013; Vachon-Presseau et al., 2016a). Hence, it has been proposed that emotional learning connects aversive life events and physical pain-chronicity (Vachon-Presseau et al., 2016b; Barroso et al., 2021). This fits well with epidemiological data revealing that early life stress correlates with chronic pain (Afari et al., 2014). Since emotional memories are crucial for posttraumatic stress disorder (PTSD), and PTSD and chronic pain share mechanisms of vulnerability and maintenance (Asmundson et al., 2002), PTSD may serve as a model for how autobiographical memory, trauma and pain interact (Siqveland et al., 2019), an intertwining also alluding to the clinical concept of *pain-proneness* (Engel, 1959).

### Linking guilt, trauma and pain

Since guilt and shame make an important part of the psychological reaction to trauma a criterion reflecting “…*persistent, distorted cognitions about the cause or consequences of the traumatic event(s) that lead the individual to blame himself/herself or others*” has been introduced into the PTSD-diagnosis in DSM-5 (American Psychiatric Association, 2013, p. 272). In Freud's concept of “moral masochism” (Engel, 1962; Freud, 1998) unconscious guilt is seen as a trauma-related maladaptive emotion demanding for reparation. Psychologically, comorbid chronic pain is then to soothe the pangs of guilt. Empirical evidence, by nature dealing with induced and hence conscious guilt so far only supports the idea that moral emotions trigger reparative behavior (Regan et al., 1972; Zhong and Liljenquist, 2006; De Hooge et al., 2011) and lead to self-denial of pleasure and even to self-punishment (Nelissen and Zeelenberg, 2009). Moreover, acceptance of electric shocks after guilt induction has been interpreted in this sense (Nelissen, 2012). Others claim that self-inflicted pain (Inbar et al., 2013) and administered painful cold-pressure-stimuli reduce guilt (Bastian et al., 2011) and even watching others in pain seems to diminish conscious feelings of guilt (Bocian and Baryla, 2020). This is noteworthy because although ample data suggest a considerable influence of *emotion on pain* (de Wied and Verbaten, 2001; Bushnell et al., 2013; Roy, 2015), still little is known about the impact of *pain on emotion* (Godinho et al., 2008; Wieser and Pauli, 2016), which surprises since depression is highly prevalent in chronic pain (Fishbain et al., 1997; Schatzberg, 2004).

### Rationale of the present study

In order to expand the state of knowledge summarized above, we tested, whether an experimental ceiling pain stimulus is apt to reduce subjective levels of guilt. Many standardized techniques like picture sets, music- and film-clips, as well as texts have been used for emotion induction in the laboratory setting (Lang et al., 2005; Coan, 2007; Gilet, 2008; Uhrig et al., 2016). Moreover, writing of an autobiographical essay has been validated with regard to emotion induction (Brewer and Doughtie, 1980; Mills and D'Mello, 2014). Since recall of individual emotional memories is highly self-referential and potentially related to the mechanisms linking emotion with trauma and pain, a written auto-bio narrative describing a serious personal moral transgression was chosen to induce guilt. A potential guilt reducing effect of suprathreshold pain-stimuli in healthy individuals was tested applying two different methods of pain induction (heat-pain vs. warmth, and cold-pressure-pain vs. lukewarm water). In a third control condition an identical heat-pain experiment was performed after induction of a neutral emotional state.

## Materials and methods

### Participants, inclusion criteria, experimental groups

All subjects gave their written informed consent as approved by the local ethics committee in accordance with the declaration of Helsinki. The order of pain application was single-blinded and randomized between test-days. Participants were mostly students recruited *via* the online platform of the University of Basel (www.markt.unibas.ch) and received a CHF 25-hourly compensation. The following inclusion criteria, were made sure of by a telephone interview: (1) Right-handedness. (2) Age between 18 and 32. (3) Native German speaker or very good command of the language. (4) Intact physical and mental health and no pain-related disorders in particular. (5) No regular intake of any medication 3 months prior to inclusion. (6) Non-smoker. (7) No cannabis consumption 3 weeks before, and during testing, and, (8) no alcohol/caffein intake 12 h before testing. Students of psychology and economy were excluded a priori. Due to substantial gender-differences in pain-perception (Riley et al., 1998; Mogil, 2012) and to safeguard against phase-specific variability with regard to the female menstrual cycle (Riley et al., 1999; De Tommaso, 2011) only male subjects were recruited. In order to study the differential impact of pain stimuli on guilt, three independent groups were recruited in a non-randomized order and tested consecutively (group A: guilt-induction/heat-pain; group B: guilt-induction/cold-pressure-pain; group C: neutral emotion induction/heat-pain) (Table 1). Testing took place in the same room using identical technical equipment between November 2016 and September 2018.

**Table 1:** Assignment to the experimental groups.

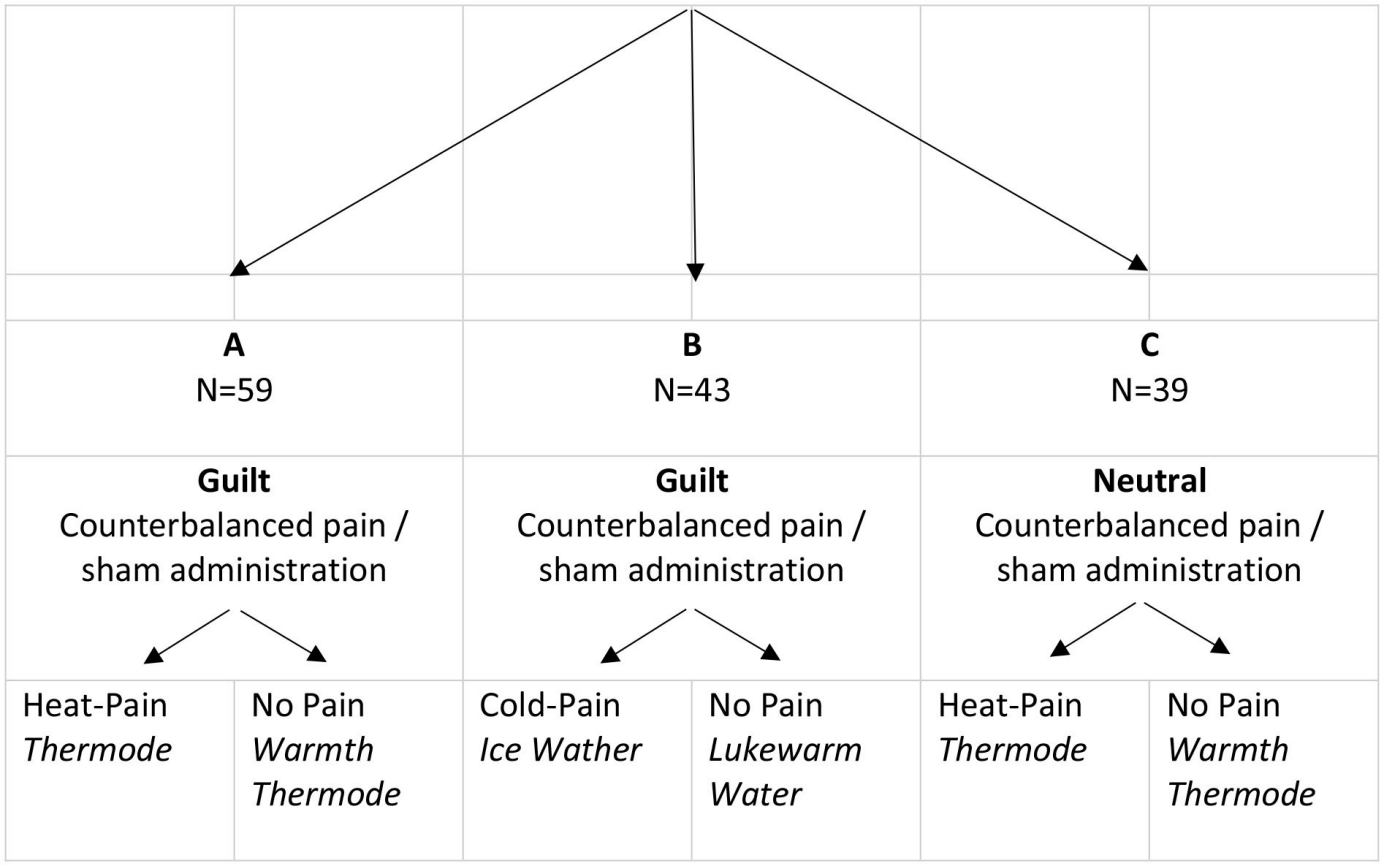

Allocation to one of the three independent groups of young healthy male participants was not randomized but within group pain/sham administration was counterbalanced over the two test-days. Groups were recruited and tested in consecutive order: Group A, B, C. Guilt was induced in group A and B on both test-days using identical procedures. In the control group C an emotionally neutral induction was performed on both test-days.

### General design and procedure

**Figure 1:**
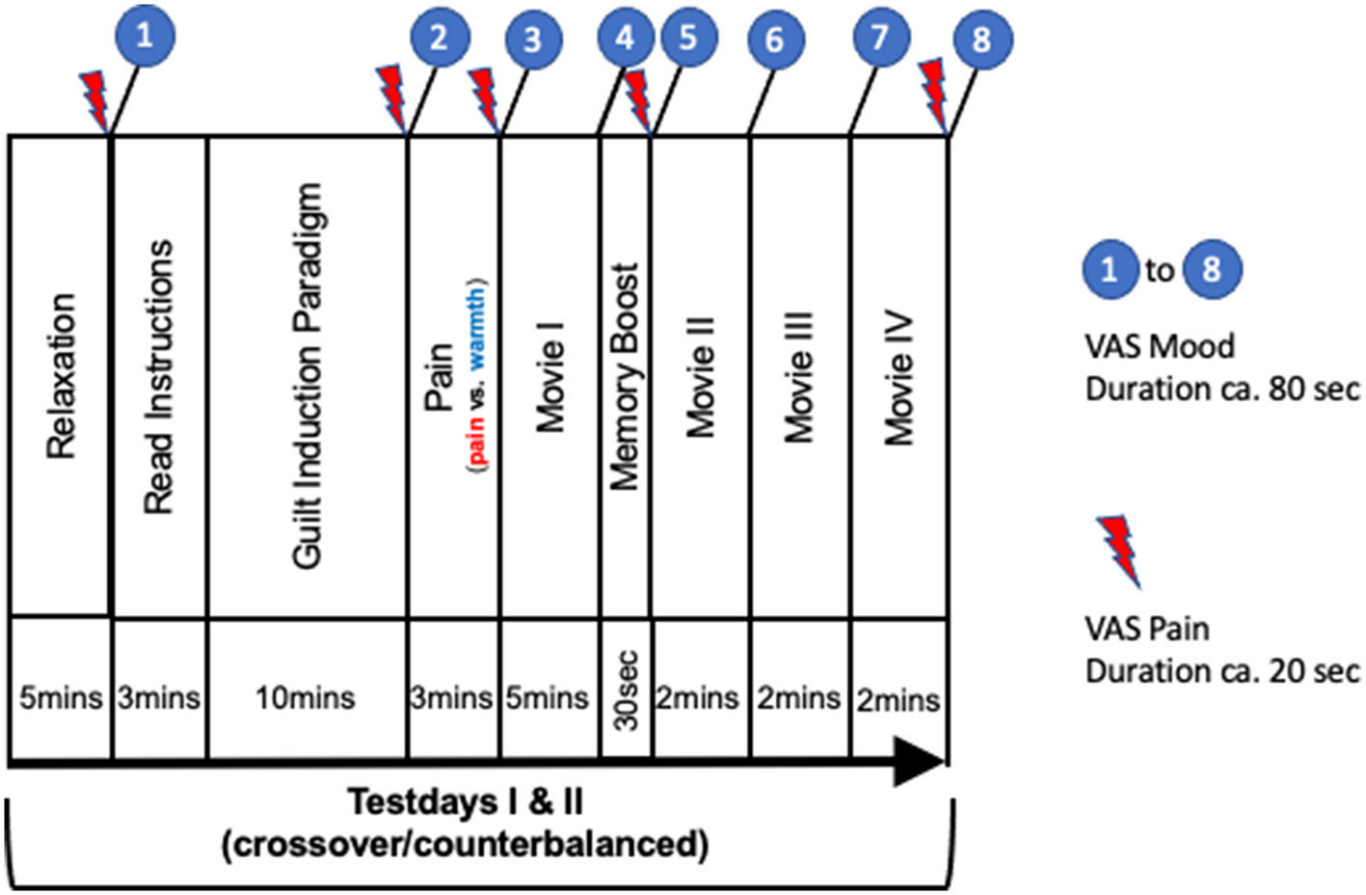
Summary of procedures. Numbers 1–8 refer to mood-state (duration ca. 80 s) and red arrows to pain recordings (duration ca. 20 s) collected using digital VAS: (1) Baseline assessments before guilt induction; (2) After reading instructions and writing of guilt-inducing essay; (3) After pain stimulation; (4) After movie I; (5) After memory boost; (6–8) After movie ll, lll, lV, respectively. In total 8 mood (e.g. I feel guilty: 0 = not at all; 10 = entirely) and 5 pain VAS ratings (0 = no pain at all−10 = maximum pain/unpleasant imaginable) were recorded throughout the experiment.

The counterbalanced cross-over design included a screening visit and two test-days 1 week apart (Figure 1). Screening and all experimental procedures took place between 9 and 11 a.m. with no intra-individual time difference between days. Verbal interaction was standardized and contact time between subject and experimenter was minimized using Presentation® software (Neurobehavioral Systems, Inc., Berkeley, CA, www.neurobs.com) for digitalization of the experimental set-up. This also allowed to control for inter-individual and between-test-day variability of timing. During oral and written instruction wording related to moral emotions was carefully avoided, and no explanation on the difference between guilt and shame was provided. In order to create an atmosphere of privacy, participants were separated from the experimenter by a partition wall.

The two test-days lasted approximately 45 min each in all of the 3 groups. Emotion induction remained identical within groups on both test-days, and pain stimuli were counterbalanced with a non-noxious warmth condition. In order to allow more recovery time, in the cold-pressure group B the memory boost was administered after movie ll.

### Screening day procedure

After completing the online questionnaires, the experimental set-up was explained and subjects were made familiar with the use of the digitalized VAS, the heat-pain stimulator (group A and C; TSA-II NeuroSensory Analyzer®, http://medoc-web.com, Ramat Yishai, Israel) and the cold-pressure procedure (group B). To quantify baseline pain perception ability, the thermal part of the quantitative sensory testing (QST) protocol (Rolke et al., 2006) was recorded in all groups, using a 9 cm^2^ standard thermode, which was attached to the left lateral calf using a blood pressure cuff pumped up to 20 mmHg. Following the validated procedure described before (Wrobel et al., 2014), the individual stimulus temperature used on test-days was determined in the heat-pain groups (A & C). Starting at 32 °C and slowly rising (1 °C / 5 s), participants had to detect the temperature intensity at which the heat-pain stimulus was rated “7” on a subjective VAS (VAS_7, ranging from 0 = no pain at all to 10 = maximum pain imaginable). The temperature corresponding to the VAS_7 intensity then served as stimulus temperature on test-days.

### Test-day procedure

#### Emotional ratings

During the two test-days, the participant's emotional state was repeatedly assessed using blocks of VAS measures (0 = not at all−10 = entirely) of nine different mood states (guilt, shame, anger at self, anger at others, anxiety, concern, empathy, happiness and balance). During each test-day a total of eight VAS rating blocks were recorded digitally (Figure 1. 1–8), each block consisting of the above nine mood ratings, which were presented on the screen in a randomized order each time. In addition, pain intensity and unpleasantness were recorded using digital VAS (0 = no pain at all; 10 = maximum pain imaginable; 0 = not unpleasant at all; 10 = maximum unpleasant imaginable) five times on each test-day (Figure 1). An emotionally neutral BBC documentary on geology was shown (except for the intro; movie I-IV, Figure 1) as a neutral distractor and filler during test-days (https://www.reddit.com/r/geology/comments/7996bv/the_mystery_of_the_egyptian_desert_glass_bbc/). In order to avoid overloading of the experiment, participants were not asked to assess the emotional valence of the movie beforehand.

#### Pain stimulation

Administration of the respective pain or sham stimuli followed a randomized and counterbalanced order. In the heat-pain condition (groups A & C) a TSA-II thermode remained attached to the right lateral calf throughout the duration of the entire experiment. Except for the 3-min stimulation, its temperature was kept at a neutral 32 °C. During this heat-pain stimulus the temperature rose immediately (5°C/s) to the VAS_7 intensity level as determined at screening. For safety reasons, it kept undulating with a frequency of 1 Hz between VAS_7 and VAS_7 - 1 ° C for three 3 min, only briefly interrupted twice for 10 s, when it dropped (5°C/s) to 32 °C. This paradigm allowed to deliver a safe as well as continuous heat-pain stimulus formally comparable to the 3 min ice water immersion in the cold-pressure condition (group B). The corresponding sham stimulus followed an identical pattern but oscillated between 39 and 38 °C during stimulation. During CPT, participants had to immerse their non-dominant (left) hand and arm into a bucket with ice–water (2–4°C) up to the elbow. The CPT sham stimulus consisted of a comparable but lukewarm water bath.

#### Guilt and neutral emotion induction

After baseline VAS assessments of emotion and pain, a standardized text appeared on a computer screen asking participants of both groups (A: heat-pain; B: cold-pressure-pain) to write about a moral transgression serious enough to hurt an important and close person in a way, which had real-life negative consequences for their relationship. In order to truly activate emotional memory content, it had to be an incident provoking intense negative feelings and emotional discomfort upon recall. To intensify this negative emotional experience, participants were asked to retrieve intimate details about the incident itself and as much background information possible on how it affected the relationship in the long run. Importantly, the instruction text avoided using any terminology alluding to morality, or guilt and shame in particular. Moreover, participants were deliberately not given any explicit information on the theoretical discrimination of guilt and shame (for detailed wording see Appendix). Given the fact, that strong negative emotional memories are surprisingly stable, participants were confronted with the identical instruction text and explicitly asked to write about the very same moral transgression also on the second test-day 1 week later (counterbalanced, cross-over design). On both test-days, participants were given 10 min to compose the paper and pencil narrative, after having carefully read the instruction shown on the screen (~3 min). In order to prevent effects of social desirability and feelings of shame and embarrassment, participants were informed in advance that their writing will be considered strictly private and they will not be asked to disclose any of its content. This statement was reiterated in the induction text (see Appendix 1). No formal specification was made regarding minimum length of the essay (e.g., word count etc.). Immediately after guilt-induction, they received a 3-min heat-pain/sham, or cold-pressure/sham stimulus, respectively. After a 5-min movie distractor (group A; movie I, Figure 1), a procedure termed “memory-boost” followed in which participants were given 30 s to mentally recall the content of the essay (for detailed wording appearing on screen see Appendix 1) after which they were asked to close their eyes and to focus particularly on emotionality. Thereafter, three further 2-min neutral movie sequences followed, being only interrupted by VAS mood rating blocks. To allow for more recovery time after the heftier CPT stimulus in group B, the memory-boost-procedure followed after movie ll.

To demonstrate that the emotion induction paradigm specifically triggers and boosts moral emotions, the identical design and formal wording was used in a control group, also stimulated with heat-pain vs. warmth (group C). Here, participants had to write about an everyday encounter with an unknown person, which was emotionally neutral and impersonal and, which also had to be recalled in a memory boost later in the experiment (for detailed wording see Appendix 2) Like in the guilt groups (A & B), the induction text was identical on both test-days). The same eight (1–8) blocks of the above nine emotional VAS ratings were recorded over the course of each test-day. Again, heat-pain and non-noxious sham stimulation were administered in a crossover and counterbalanced manner immediately after the induction procedure.

### Psychological measures

To characterize participants with regard to their mental health status and their perception of moral emotions, participants had to complete an electronic survey (https://www.soscisurvey.de®) at screening, which consisted of questionnaires related to trauma [Childhood Trauma Questionnaire, CTQ (Wingenfeld et al., 2010), Posttraumatic Diagnostic Scale, PDS (Griesel et al., 2006)], moral emotions [Test of Self-Conscious Affect, TOSCA (Tangney et al., 2000; Rusch et al., 2007), Personal Feelings Questionnaire, PFQ (Harder and Greenwald, 1999; Rusch et al., 2007)], depression [Beck Depression Inventory, BDI (Beck and Hurvich, 1959; Kuhner et al., 2007)], and anxiety, [Trait Anxiety Inventory, STAI (Laux et al., 1981)]. Based on the idea that moral attitude as well as behavior are linked to religious believes and practices, subjects indicated whether they consider themselves religious on a computerized visual analog scale [(VAS: 0 = not religious at all, and 10 = most strongly religious].

The Questionnaires related to trauma (CTQ, PDS), depression (BDI) and anxiety (STAI State) were used to screen for clinically relevant symptoms. Foa (Foa et al., 1997) established guidelines, according to which a PTDS total score below 10 represents mild, 11–20 moderate, 21–35 moderate to severe, and above 35 severe PTSD. Giesbrecht (Giesbrecht et al., 2007) studied 185 (146 females) undergraduates and described average CTQ total scores of 34.06 (SD 10.54). For the Beck Depression Inventory, it is generally agreed that sum scores <14 are normal (Kuhner et al., 2007), and the study of Ercan (Ercan et al., 2015) described a cut off value of 44 for the STAI state measure. These values were used as a guideline for the assessment (see Results).

### Statistical analyses

All statistical analyses of behavioral data were performed in R (http://www.r-project.org/) and in SPSS 25 for Mac. Linear mixed models (nlme-package) combined with ANOVA (SS II) were applied. Participant ID was included as the random effect of the mixed model.

#### Manipulation checks

First, we analyzed whether the heat pain and CPT stimulation was sufficiently painful and unpleasant by calculating separate linear models for each rating within both pain induction groups separately (group A & C: heat-pain vs. warmth; group B: cold-pressure-pain vs. warmth). Dependent variables were the subjective VAS assessments of pain intensity and unpleasantness as recorded immediately after pain/warmth induction (timepoint 3, Figure 1), while the pain-condition was the independent variable. Then we tested whether guilt induction was successful and limited to moral emotions (timepoint 2, Figure 1). Dependent variables were the subjective emotion ratings (guilt, shame, anger at self, anger at others, anxiety, concern, empathy, happiness, balance). Independent variables were the factors time (before and after guilt induction) and pain-condition for which also an interaction term (time^*^pain-condition) was included. Here, we were interested in the main effect of time.

#### Analysis of the effect of the pain stimuli on emotion ratings using VAS

To assess the effect of heat- and cold-pressure-pain on perceived emotion immediately after the stimulus and beyond, we analyzed whether participants' VAS ratings differed after receiving a warmth or a pain stimulus. For each of the ratings, a separate linear model was calculated. Dependent variables were the nine respective emotional ratings per block and over time [after pain / sham stimulation (3); before the memory boost (4); after the memory boost (5); after movie II (6); after movie III (7); after movie IV (8)] in each group separately. Pain-condition served as an independent variable. Baseline emotional ratings, i.e., ratings before guilt induction, and age were included as covariates. Here, we were interested in the main effect of pain-condition (heat-pain or CPT, respectively, vs. sham).

#### Correction for multiple testing and effect sizes

Since guilt constituted the primary outcome variable, the threshold for this rating was set to *p* < 0.05. For the eight remaining ratings, Bonferroni correction for eight independent tests revealed a threshold at *p* < 0.00625. Effect sizes calculated for repeated-measurement factors are influenced by the correlation between the repeated measures. Hence, they are not comparable to effect sizes for factors used in between-subject designs. To provide a measure comparable to the effect sizes of between-subject designs (Jaeger et al., 2017), we calculated generalized semi-partial *R*^2^ ($R_{\text{β}*}^{2}$) (Nakagawa and Schielzeth, 2013). For easier interpretability, we report *r* computed by the square root of $R_{\text{β}*}^{2}$ with r = 0.1 = small, 0.3 = intermediate, 0.5 = large effect.

#### Sample comparison

To test for differences between both essay conditions (guilt vs. neutral), a between-group comparison of all emotional ratings after emotion-induction and after memory-boost was performed. Dependent variables were the guilt ratings after emotion-induction and after the memory-boost. Pain- and essay condition (guilt vs. neutral) served as independent variables. Again, baseline guilt ratings and age were included as covariates. The following model was applied: VAS emotion 2 ~ VAS emotion 1 + AGE + factor (pain-condition) + factor (group).

To test whether there is an order effect for guilt induction within subjects (test-day 1 vs. test-day 2) a pilot study, with 42 undergraduate students (22 females) was performed, where instead of pain/sham a 0-back attention test was administered immediately after guilt induction (time 2, Figure 1) on both days. Moreover, the actual data of group A and B were analyzed with regard to test-days, i.e., irrespective of counterbalancing to pain/sham. Repeated measure ANOVAs were performed and sex as well as age were included as covariates. Additionally, between-group measures of questionnaires, QST and VAS_7 in °C were compared using ANOVA.

## Results

### Participants

#### Group characteristics

Each group was recruited independently without random assignment of the participants: Group A (heat-pain/guilt induction) 59 males, mean-age 23.44, range 18–31, 95% CI 22.49–24.39 years. Group B (cold-pressure-pain/guilt induction) 43 males, mean-age 23.14, range 18–29, 95% CI 22.14–24.14 years. Group C (heat-pain and neutral induction) 39 males, mean-age 23.51, range 18–30, 95% CI 22.45–24.58 years. A one-factorial ANOVA did not reveal any difference in age between groups.

Questionnaires did not disclose any differences between groups except for CTQ ratings, which showed a higher total score in group A (*post-hoc* Bonferroni correction: *p* = 0.008). PDS total scores were below 10 in all groups, and the range of CTQ total score was between 39.07 and 45.63 across groups. BDI scores were below 6 and scores for STAI state below 39.1 (for cut off values based on the literature, see the methods section). Based on these measures, and on the structured telephone-interview, where a thorough medical and psychiatric history was obtained prior to inclusion, no symptomatology of clinical relevance could be described in our population. For an overview see Table 2.

**Table 2:** Questionnaires and QST pain-thresholds.

**Measure**	**Guilt heat-pain (group A)** **(*N* = 59)**	**Guilt CPT pain (group B)** **(*N* = 43)**	**Neutral (group C)** **(*N* = 39)**	**Test-value**	***p*-value**
CTQ total score	45.63 ± 12.35	39.07 ± 9.42	40.51 ± 9.02	*F*_(2;138)_ = 5.399	<0.01
PDS total score	9.07 ± 2.34	9.40 ± 2.97	9.82 ± 2.68	*F*_(2;138)_ = 0.956	ns
BDI sum score	5.85 ± 4.52	5.63 ± 3.79	5.67 ± 4.49	*F*_(2;138)_ = 1.085	ns
STAI state total score	39.10 ± 7.93	37.05 ± 7.42	37.62 ± 8.76	*F*_(2;138)_ = 0.901	ns
Religion (VAS 0–10)	26.61 ± 32.34	27.30 ± 31.98	27.54 ± 29.43	*F*_(2;138)_ = 0.012	ns
TOSCA guilt	43.53 ± 5.17	44.14 ± 4.63	44.18 ± 4.38	*F*_(2;138)_ = 0.299	ns
TOSCA shame	28.22 ± 6.49	27.14 ± 5.99	27.44 ± 7.16	*F*_(2;138)_ = 0.376	ns
PFQ guilt-prone	6.29 ± 3.24	7.02 ± 3.40	6.87 ± 3.23	*F*_(2;138)_ = 0.720	ns
PFQ shame prone	7.97 ± 4.31	8.02 ± 4.55	7.85 ± 3.70	*F*_(2;138)_ = 0.019	ns
CPTh Mean ± SD °C	14.53± 9.38	10.15 ± 7.73	15.61 ± 8.63	*F*_(2;139)_ = 4.720	<0.05
HPTh Mean ± SD °C	45.68 ± 2.57	46.23 ± 2.98	46.11 ± 1.78	*F*_(2;138)_ = 0.828	ns
VAS_7 °C	47.79 ± 1.24	na	47.82 ± 1.46	*F*_(1;96)_ = 0.014	ns

Questionnaires and Perceptional Characteristics: Questionnaire results (CTQ, Childhood Trauma Questionnaire; PDS, Posttraumatic Diagnostic Scale; BDI, Beck Depression Inventory; STAI, State Trait Anxiety Inventory; Religiosity; TOSCA, Test of Self-Conscious Affect; PFQ, Personal Feelings Questionnaire) and thermal QST (quantitative sensory testing) recordings (CPTh, cold-detection-threshold; HPTh, heat-pain-threshold)are shown as mean ± SD. Group comparison was calculated using one factorial ANOVA with Bonferroni correction.

### Manipulation checks

#### Effectiveness of pain stimuli

Baseline heat-pain threshold as mirrored by QST temperature measures at screening did not differ among the three groups. In all groups, pain stimulation yielded a strong effect. Accordingly, VAS intensity and unpleasantness ratings significantly differed when compared to the respective within-group sham condition [group A: intensity *t*_(58)_ = 23.54, *p* < 0.0001, *r* = 0.91, unpleasantness *t*_(58)_ = 20.69, *p* < 0.0001, *r* = 0.89; group B: intensity *t*_(42)_ = 21.4, *p* < 0.0001, *r* = 0.92, unpleasantness *t*_(42)_ = 17.8, *p* < 0.0001, *r* = 0.87; group C: intensity *t*_(38)_ = 11.57, *p* < 0.0001, *r* = 0.80, unpleasantness: *t*_(38)_ = 10.49, *p* < 0.0001, *r* = 0.76]. Between group comparison of pain intensity and unpleasantness VAS ratings using ANOVA showed significant differences [intensity: *F*_(2;138)_ = 3.12, *p* = 0.047; unpleasantness: *F*_(2;138)_ = 4.83, *p* = 0.009] with the highest ratings (mean ± SD) in the CPT group (group B: intensity 7.69 ± 2.01; unpleasantness 7.99 ± 2.01), and the lowest in the control group (C: intensity 6.49 ± 2.66; unpleasantness 6.51 ± 2.74), whereas the ratings in group A were intermediate (A: intensity 7.26 ± 2.02; unpleasantness 7.12 ± 1.86). After Bonferroni correction, only the differences between the CPT and the control group (intensity *p* = 0.044; unpleasantness *p* = 0.008) remained significant.

#### Effectiveness of guilt induction

Immediately after guilt induction, subjects in group A (heat-pain/guilt) perceived significantly more guilt [*t*_(175)_ = −16.91, *p* < 0.0001, *r* = 0.70], shame [*t*_(175)_ = −15.01, *p* < 0.0001, *r* = 0.64], concern [*t*_(175)_ = −11.03, *p* < 0.0001, *r* = 0.53], empathy [*t*_(175)_ = −2.90, *p* = 0.0.004, *r* = 0.17], and anger at themselves [t_(175)_ = −11.69, *p* < 0.0001, *r* = 0.54], as well as more anger at others [*t*_(175)_ = −3.86, *p* = 0.0002, *r* = 0.22]. Accordingly, they felt substantially less balanced [*t*_(175)_ = 9.24, *p* < 0.0001, *r* = 0.43], and happy [*t*_(175)_ = 8.88, *p* < 0.0001, *r* = 0.41], but there was no significant change in felt anxiety [*t*_(175)_ = −2.20, *p* = 0.029, *r* = 0.12] after guilt induction. Moreover, no significant time * pain-condition interaction (guilt *p* = 0.24, all other emotion ratings *p's* >0.18) or main effect of pain-condition on any emotion rating could be detected (guilt *p* = 0.69, all other *p's* >0.013), demonstrating that guilt, as well as all other emotional ratings did not differ between counterbalanced conditions on test days (heat-pain vs. warmth).

In group B (CPT / guilt), subjects also perceived significantly more guilt [*t*_(125)_ = −13.63, *p* < 0.0001, *r* = 0.67], shame [*t*_(125)_ = −14.01, *p* < 0.0001, *r* = 0.69], concern [*t*_(125)_ = −9.46, *p* < 0.0001, *r* = 0.55] and anger at themself [*t*_(125)_ = −10.98, *p* < 0.0001, *r* = 0.59], and they were also less balanced [*t*_(125)_ = 9.24, *p* < 0.0001, *r* = 0.43] and less happy [*t*_(125)_ = 7.43, *p* < 0.0001, *r* = 0.45]. However, there was no significant change in felt anxiety [*t*_(125)_ = −1.66, *p* = 0.10, *r* = 0.11], empathy [*t*_(125)_ = −1.99, *p* = 0.049, *r* = 0.13], or anger at others [*t*_(125)_ = −2.55, *p* = 0.012, *r* = 0.17]. For guilt, a significant time * pain-condition interaction was found (*F*_(1,124)_ = 5.78, *p* = 0.018, *r* = 0.67] indicating that on test-days with sham stimulation (lukewarm water exposition), guilt induction was more pronounced [*t*_(42)_ = −12.55, *p* < 0.00001, *r* = 0.77] than on cold-pressure days [*t*_(41)_ = −6.92, *p* < 0.00001, *r* = 0.55]. No other significant interactions (all *p's* >0.27), or main effects of pain-condition were found (guilt *p* = 0.66, all other *p* > 0.04).

#### Effectiveness of guilt reminder (“memory boost”)

In group A, the guilt reminder considerably increased the subjects perceived guilt [*t*_(175)_ = −12.66, *p* < 0.0001, *r* =0.57], shame [*t*_(175)_ = −13.12, *p* < 0.0001, *r* = 0.58], and concern [*t*_(175)_ = −11.75, *p* < 0.0001 *r* = 0.53]. They also felt more anger at themselves [*t*_(175)_ = −13.14, *p* < 0.0001, *r* = 0.57], and at others [*t*_(175)_ = −4.57, *p* < 0.0001, *r* = 0.25], as well as more anxiety [*t*_(175)_ = −4.16, *p* = 0.0001, *r* = 0.21]. Furthermore, they felt less balanced [*t*_(175)_ = 9.24, *p* < 0.0001, *r* = 0.43], and less happy [*t*_(175)_ = 8.88, *p* < 0.0001, *r* = 0.58]. However, there was no significant change in felt empathy [*t*_(175)_ = −2.43, *p* = 0.016, *r* = 0.13], and there was no time * pain-condition interaction (guilt *p* = 0.09, all other *p* > 0.09), nor was there a main effect of pain-condition on any of the emotion ratings after the guilt reminder (guilt *p* = 0.39, all other *p* > 0.09).

In group B, subjects felt significantly more guilty [*t*_(125)_ = −9.71, *p* < 0.0001, *r* = 0.54], ashamed [*t*_(125)_ = −10.01, *p* < 0.0001, *r* = 0.54], concerned [*t*_(125)_ = −7.97, *p* < 0.0001, *r* = 0.46], and empathic [*t*_(125)_ = −3.15, *p* = 0.002, *r* = 0.18]. Moreover, they were more angry at themself [*t*_(125)_ = −10.9, *p* < 0.0001, *r* = 0.56], and at others [*t*_(125)_ = −2.89, *p* = 0.005, *r* = 0.17], as well as more anxious [*t*_(125)_ = −3.71, *p* = 0.0001, *r* = 0.21) after the memory boost. Also, they were less balanced [*t*_(125)_ = 5.38, *p* = 0.0001, *r* = 0.32], and less happy [*t*_(125)_ = 5.78, *p* = 0.0003, *r* = 0.33]. Furthermore, no significant time * pain-condition interaction (guilt *p* = 0.95, all other *p* > 0.55), nor a main effect of pain-condition was found on any emotion rating after reinduction of guilt (guilt *p* = 0.20, all other *p* > 0.04).

**Figure 2:**
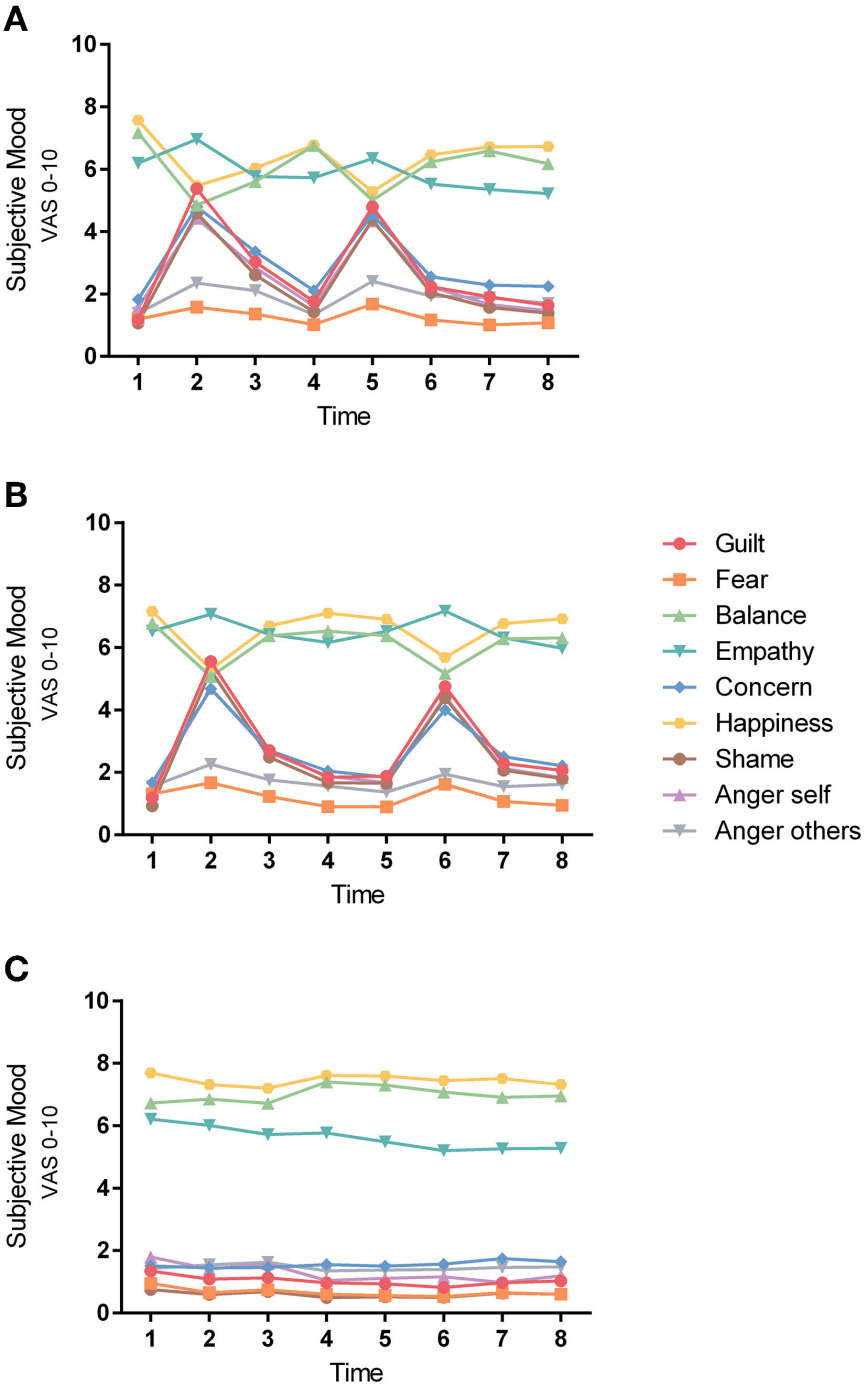
Course of all emotions. All of the 9 digital VAS emotion ratings for the 3 experimental groups **(A)** guilt-induction/heat-pain; **(B)** guilt-induction/cold-pressure-pain; **(C)** neutral emotion induction/heat-pain are shown. Data of both test-days were collapsed, and means of emotional ratings are shown. Hence, there is no split between pain/sham.

Between-group analyses (see methods) revealed a group main effect (guilt-induction vs. neutral) for all emotional ratings after emotion induction, as well as after the memory boost (all *p's* < 0.003). At both timepoints, higher ratings were found in the guilt groups for guilt, shame, concern, empathy, anger at themselves, and anger at others, as well as lower ratings for feeling balanced and happiness when compared to the neutral induction (see also Figure 2C).

In the pain-free guilt-induction-pilot no main-effect of test-day and no interaction with age or sex could be demonstrated (analyzes and graphs not shown). Moreover, data of group A and B reanalyzed without counterbalancing did also not reveal a main-effect of test-day (analyzes and graphs not shown). Hence, we conclude that strength of guilt induction does not fade on test-day two.

### Effect of pain-condition on emotional ratings

Generally, no evidence was found for an effect of experimental pain on emotion.

#### Group A (heat-pain/sham; guilt induction)

**Figure 3:**
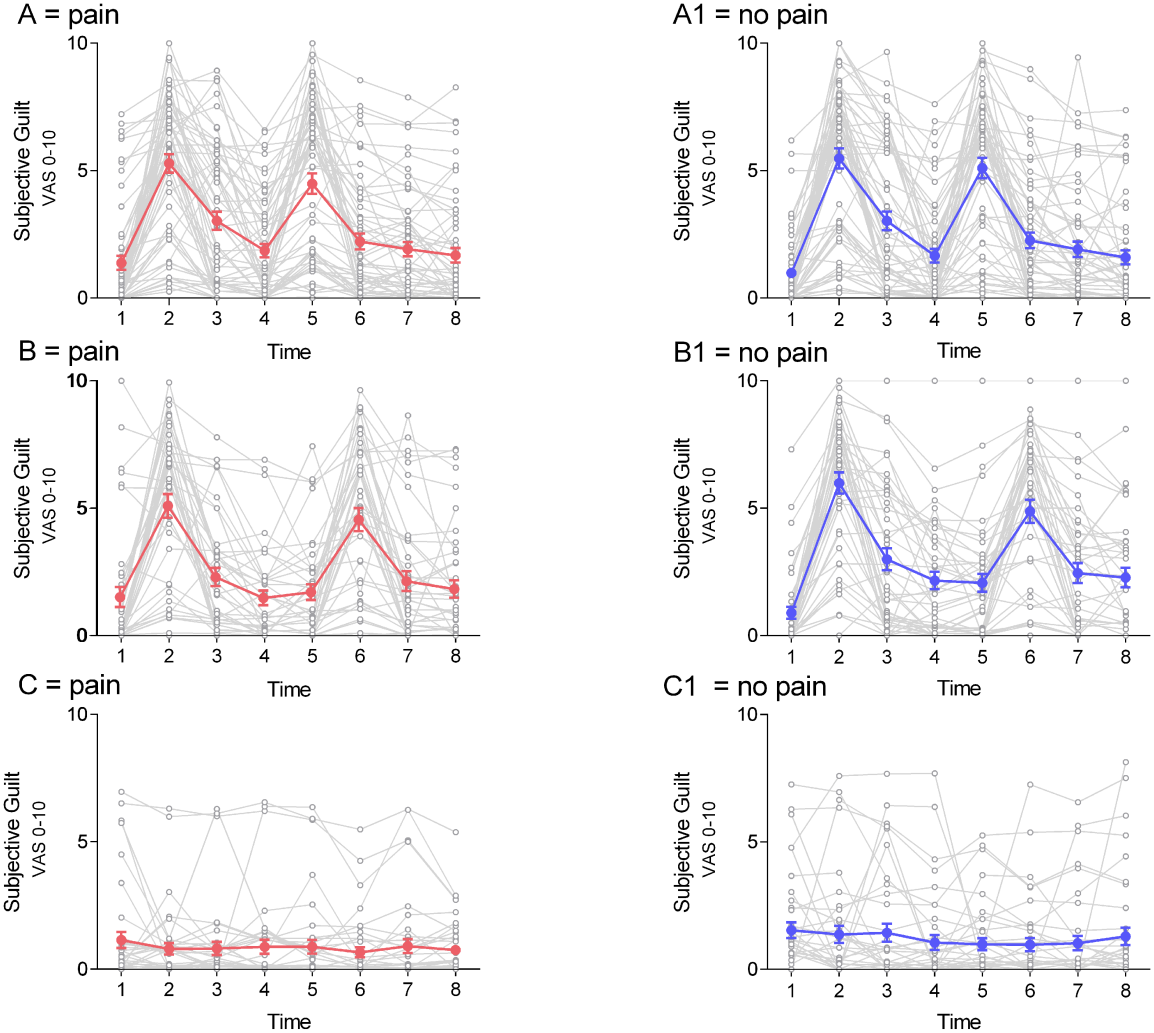
Course of guilt on test-days. Data of individual guilt ratings (mean ± sem) are shown over time in all 3 groups **(A)** guilt-induction/heat-pain; **(B)** guilt-induction/cold-pressure-pain; **(C)** neutral emotion induction/heat-pain) for both test-days. Guilt in the supra-threshold pain-condition is shown in red **(A–C)**. The guilt line of the respective sham condition is shown in blue **(A1–C1)**.

When compared to sham, the heat-pain stimulus had no significant effect on emotional ratings during the entire duration of the experiment (numbers in brackets represent points in time as indicated in Figure 1): (3) guilt immediately after pain/sham stimulation: *t*_(75)_ = 0.32, *p* = 0.75, *r* = 0.03, *p* of all other emotion ratings >0.01, all *r* < 0.21; (4) guilt before memory boost (after movie I): *t*_(75)_ = 1.01, *p* = 0.3, *r* = 0.07, *p* of all other emotion ratings >0.04, all *r* < 0.14; (5) guilt after the memory boost: *t*_(75)_ = −1.34, *p* = 0.19, *r* = 0.12, *p* of all other emotion ratings >0.03, all *r* < 0.16; (6) guilt after movie II: *t*_(75)_ = 0.08, *p* = 0.94, *r* = 0.01, *p* of all other emotion ratings >0.09, all *r* < 0.12; (7) guilt after movie III: *t*_(75)_ = 0.24, *p* = 0.81, *r* = 0.02, *p* of all other emotion ratings >0.02, all *r* < 0.13; and (8) guilt after movie IV: *t*_(75)_ = 0.5, *p* = 0.62, *r* = 0.03, *p* all other emotion ratings >0.15, all *r* < 0.12). For guilt ratings during the experiment see Figure 3.

#### Group B (cold-pressure-pain/sham; guilt induction)

No significant effect of cold-pressure-pain on emotional ratings at any time was found: (3) guilt immediately after CPT / sham stimulation: *t*_(40)_ = −0.9, *p* = 0.38, *r* = 0.08, *p* of all other emotion ratings >0.09, all *r* < 0.17; (4) guilt after movie I: guilt *t*_(40)_ = −1.38, *p* = 0.17, *r* = 0.0.12, *p* of all other emotion ratings >0.21, all *r* < 0.11; (5) guilt before memory boost (after movie II): guilt *t*_(40)_ = −1.01, *p* = 0.32, *r* = 0.09, *p* of all other emotion ratings >0.03, all *r* < 0.16; (6) guilt after memory boost: guilt *t*_(39)_ = 0.73, *p* = 0.47, *r* = 0.07, *p* of all other emotion ratings >0.29, all *r* < 0.11; (7) guilt after movie III: guilt *t*_(39)_ = −0.2, *p* = 0.84, *r* = 0.02, *p* of all other emotion ratings >0.17, all *r* < 0.13; and (8) guilt after movie IV: *t*_(39)_ = −0.72, *p* = 0.48, *r* = 0.06, *p* all other emotion ratings >0.11, all *r* < 0.13). Figures 3B,B1 depict guilt-ratings in the cold-pressure group.

#### Group C (control sample heat-pain/sham, with neutral induction)

Within-group analysis of all respective emotional ratings after the neutral induction paradigm did not reveal any significant changes after the heat-pain stimulus [guilt *t*_(115)_ = 1.14, *p* = 0.25, *r* = 0.08, *p* of the remaining: *p* > 0.09, all *r* < 0.12], nor after the memory boost procedure [guilt *t*_(115)_ = −0.20, *p* = 0.84, *r* = 0.01, *p* of the remaining emotion ratings: *p* > 0.29, all *r* < 0.07]. Moreover, there was no significant effect of time, nor was there a time*pain-condition interaction.

## Discussion

The present study pursued the goal of testing whether different supra-threshold pain stimuli are apt to impact moral emotions in physically and mentally healthy individuals. Although guilt-induction using recall of autobiographical memory content worked reliably, no empirical evidence could be found on a behavioral level, i.e., no tangible decrease of guilt, nor any influence on concomitant positive or negative emotions could be captured after pain-stimulation when compared to the respective non-noxious sham condition. In this respect, our results differ from those of earlier empirical work. However, several methodological aspects need critical consideration.

### The role of the pain stimuli

Given the consistently large effects on VAS (0–10) pain ratings for intensity (group A: *r* = 0.91; group B: *r* = 0.92; group C: *r* = 0.80) and unpleasantness (group A: *r* = 0.89; group B: *r* = 0.87; group C: *r* = 0.76) immediately after administration of pain, it is highly unlikely that the used ceiling heat-pain and ice-water stimuli were insufficient. Even the considerably painful and unpleasant cold-pressure stimulus showed no influence on the perception of guilt, and previous findings based on comparable methodology could not be replicated (Bastian et al., 2011). However, it cannot be finally excluded that the supra-threshold pain stimuli were too strong in the sense that they acted as an unspecific distractor, masking the actual desired effect by not only abruptly withdrawing attention from the cognitive (Crombez et al., 1998), but also from the emotional focus on guilt. This phenomenon could have hindered the pain to exert the hypothesized specific influence on negative moral emotions in both experimental groups (A & B), in which pain intensity and pleasantness ratings did not differ statistically.

In contrast, there was a difference in subjective VAS pain ratings between the cold-pressure (B, with guilt-induction) and the control group (C, without guilt-induction). Because heat-pain was also administered in group A and individual differences in pain-threshold were accounted for, the difference cannot be simply explained by the weaker heat-pain stimulus and its application to the lower limb, where thresholds are generally higher (Rolke et al., 2006) and the stimulus area was comparably small (9 cm^2^). Therefore, the considerably higher unpleasantness of perceived pain in the cold-pressure group B can only be partly explained by the sheer strength of the ice-water stimulus itself, and thus must also be attributed to the formative influence of the preceding negative emotional state elicited by guilt induction. Implicitly, this interpretation corroborates the effect of the auto-biographical method and points to a possible influence of emotion on pain perception.

### The role of guilt induction

The presented data unequivocally show that a written auto-bio-graphical essay is apt to elicit guilt as measured on a behavioral level using a visual analog scale. Statistically, the respective effect was strong in both of the guilt groups (A & B) on both test-days, showing that the paradigm can be used in a crossover design. The same effect was demonstrated for the “memory-boost”, which was performed additionally on both days later during the experiment. This demonstrates that recall of relevant emotional biographical content represents an internal stimulus, that is highly efficient in inducing moral emotions. Since the effect did not significantly fade upon repetition on a second test-day, the auto-biographical method might even be superior to standardized external stimuli, being commonly used for mood induction. Furthermore, we replicated previous data by showing that the autobiographical recall was not specific for the target emotion (Mills and D'Mello, 2014). Incidentally, a variety of additional negative mood states like shame, concern, and anger were triggered. This can be partially explained by the fact that subjects were not provided with any theoretical explanation on how to distinguish guilt from shame beforehand. At the same time, the concomitant positive emotions like feeling happy and being balanced were significantly reduced, which again confirms the inner coherence of the method.

### Limitations

The groups were recruited consecutively and there was no randomization at group level. This was mainly due to the rather complex design and the respective technical effort, which considerably differed among groups.

For ethical reasons, participants were not asked to disclose the content of their writing, nor were the moral terms guilt and shame explicitly mentioned in the induction text (see Appendix 1). From a methodological perspective, this approach appears to be a double-edged sword. Deliberately protecting the intimacy of participants might have enhanced their willingness to write about a truly embarrassing moral transgression. However, by not having to disclose the content of their writing, subjects escaped the imaginary threat of social judgment, possibly mitigating the emotional power of morality. Moreover, no formal instructions were given regarding the auto-bio-essay (e.g., word count etc.), further precluding to characterize the written text, neither with regard to its content, nor formally. However, in a sub-sample of 20 participants of group A, a *post-hoc* semi-structured interview was conducted no later than 6 weeks after the experiment. This covered biographical contents like early bonding, upbringing, social circumstances, as well as former and current relationships. Additionally, participants were given the opportunity to report on the moral transgression recalled and described in the experimental situation. This part, separately approved by the local ethics committee, was strictly voluntary, and all of the above 20 subjects participated. Qualitative analyses of the interview transcripts revealed unambiguous evidence of the subjective perception of guilt and shame by the participants (Goetzmann et al., in preparation), hence confirming the quantitative findings.

It is important to mention that the lack of specificity of emotion induction with comparable ratings of guilt and shame might have interfered with the hypothesis under investigation. However, in a healthy population, feelings of guilt and shame may generally be associated with also experiencing other emotions rather than being strictly isolated affects. Finally, it may be criticized that no validated ratings of the movie sequences shown throughout out the experiment on both test-days are available.

## Conclusion and further implications

To our knowledge, this is the first study to describe the induction of moral emotions by means of a written autobiographical essay using a fully digitalized and time-critical paradigm lasting over 45 min. We were not only able to confirm its high effectiveness in inducing the target moral emotion of guilt, but could also capture the concomitant positive and negative mood states during the entire experiment. Since the method involved emotional memories, it was possible to reliably trigger, boost, re-trigger, and re-boost mood states on two consecutive test-days 1 week apart. Thus, the applied mood induction procedure appears to be particularly suited for cross-over designs.

However, although guilt induction was highly effective and the pain stimuli were sufficiently strong, we were not able to replicate previous work based on similar methods claiming to empirically capture respective effects (Bastian et al., 2011; Inbar et al., 2013). In this regard, we agree with Prager and colleagues (Prager et al., 2019) that reporting negative results is of utmost importance, because it puts earlier data in the field into perspective (Zhong and Liljenquist, 2006; De Hooge et al., 2007, 2011; De Hooge, 2012).

Based on our findings, we critically state that the interrelation of pain and moral emotions might be technically more difficult to address than expected. In particular, when considering the fact, that negative moral emotions are not a straightforward conscious cognitive phenomenon and hence, are hard to map technically. Future research should investigate patient populations with chronic wide-spread pain and a history of trauma using comparable designs augmented by psycho-physiological methods. More sophisticated techniques like heart rate variability (HRV) and cortisol measures should be used to provide deeper insight into the intricate mechanisms of unconscious phenomena, which are likely to resonate in the autonomous nervous system and thus are beyond the reach of simple behavioral assessment.

Furthermore, it is highly likely that patients show stronger behavioral effects with regard to emotion induction and pain modulation. Also, topics like location of pain infliction, as well as varying pain intensities and qualities must be addressed empirically. Moreover, it should be investigated if results differ depending on whether pain is externally administered or self-inflicted. Finally, our findings suggest that it is challenging or even impossible to translate a hypothesis exclusively derived from clinical observations (Engel, 1959; Adler et al., 1989; Freud, 1998) to an experimental set-up and to a healthy population.

## Data availability statement

The raw data supporting the conclusions of this article will be made available by the authors, without undue reservation.

## Ethics statement

The studies involving human participants were reviewed and approved by all subjects gave written informed consent. The study was approved in compliance with the Declaration of Helsinki by the Local Ethics Committee Northwest/Central Switzerland (EKNZ; registration number: BASEC 2015-00184; short title PG_2015). The patients/participants provided their written informed consent to participate in this study.

## Author contributions

The study was designed by KSc, NS, DQ, LW, and LG. KSc wrote the ethics proposal. KSc, NS, SS, JE, and AV were responsible for recruitment of participants and data collection. Data analysis was completed by NS, KSc, AV, JE, and SS. AV made the graphs. KSc, AW, JE, and NS wrote the manuscript draft. MgH, DQ, NS, NE, AW, and KSt thoroughly revised the manuscript. All of the authors read and approved the final manuscript. All authors contributed to the article and approved the submitted version.

## Funding

The study was supported by the Division of Cognitive Neuroscience, University of Basel, Switzerland and by the research fund of the Psychiatric University Clinics (UPK), Basel, Switzerland.

## Conflict of interest

The authors declare that the research was conducted in the absence of any commercial or financial relationships that could be construed as a potential conflict of interest.

## Publisher's note

All claims expressed in this article are solely those of the authors and do not necessarily represent those of their affiliated organizations, or those of the publisher, the editors and the reviewers. Any product that may be evaluated in this article, or claim that may be made by its manufacturer, is not guaranteed or endorsed by the publisher.

## References

[B1] Adler R. H. Zlot S. Hurny C. Minder C. (1989). Engel's “Psychogenic Pain and the Pain-Prone Patient:” a retrospective, controlled clinical study. Psychosom. Med. 51, 87–101. 10.1097/00006842-198901000-00009 2648449

[B2] Afari N. Ahumada S. M. Wright L. J. Mostoufi S. Golnari G. Reis V.. (2014). Psychological trauma and functional somatic syndromes: a systematic review and meta-analysis. Psychosom. Med. 76, 2–11. 10.1097/PSY.0000000000000010 24336429 PMC3894419

[B3] American Psychiatric Association (2013). Diagnostic and Statistical Manual of Mental Disorders. 5th ed. Washington, DC. 10.1176/appi.books.9780890425596

[B4] Asmundson G. J. Coons M. J. Taylor S. Katz J. (2002). PTSD and the experience of pain: research and clinical implications of shared vulnerability and mutual maintenance models. Can. J. Psychiatry. 47, 930–937. 10.1177/070674370204701004 12553128

[B5] Barroso J. Branco P. Apkarian A. V. (2021). Brain mechanisms of chronic pain: critical role of translational approach. Transl. Res. 238, 76–89. 10.1016/j.trsl.2021.06.004 34182187 PMC8572168

[B6] Bastian B. Jetten J. Fasoli F. (2011). Cleansing the soul by hurting the flesh: the guilt-reducing effect of pain. Psychol. Sci. 22, 334–335. 10.1177/0956797610397058 21245493

[B7] Beck A. T. Hurvich M. S. (1959). Psychological correlates of depression. 1. Frequency of masochistic dream content in a private practice sample. Psychosom. Med. 21, 50–55. 10.1097/00006842-195901000-00007 13634302

[B8] Bocian K. Baryla W. (2020). Pain(less) cleansing: Watching other people in pain reduces guilt and sadness but not shame. PloS ONE. 15, e0244429. 10.1371/journal.pone.0244429 33378345 PMC7773247

[B9] Brewer D. Doughtie E. B. (1980). Induction of mood and mood shift. J. Clin. Psychol. 36, 215–226. 7391236 10.1002/1097-4679(198001)36:1<215::aid-jclp2270360127>3.0.co;2-6

[B10] Bushnell M. C. Ceko M. Low L. A. (2013). Cognitive and emotional control of pain and its disruption in chronic pain. Nat. Rev. Neurosci. 14, 502–511. 10.1038/nrn3516 23719569 PMC4465351

[B11] Coan J. (2007). Handbook of Emotion Elicitation and Assessment Oxford: Oxford university press.

[B12] Crombez G. Eccleston C. Baeyens F. Eelen P. (1998). Attentional disruption is enhanced by the threat of pain. Behav. Res. Ther. 36, 195–204. 10.1016/S0005-7967(97)10008-0 9613025

[B13] De Hooge I. E. (2012). IE. The exemplary social emotion guilt: Not so relationship-oriented when another person repairs for you. Cogn. Emot. 26, 1189–1207. 10.1080/02699931.2011.640663 22394129

[B14] De Hooge I. E. Nelissen R. Breugelmans S. M. Zeelenberg M. (2011). What is moral about guilt? acting “prosocially” at the disadvantage of others. J. Pers. Soc. Psychol. 100, 462. 10.1037/a0021459 21244173

[B15] De Hooge I. E. Zeelenberg M. Breugelmans S. M. (2007). Moral sentiments and cooperation: Differential influences of shame and guilt. Cogn. Emot. 21, 1025–1042. 10.1080/02699930600980874

[B16] De Tommaso M. (2011). Pain perception during menstrual cycle. Curr. Pain Headache Rep. 15, 400–406. 10.1007/s11916-011-0207-1 21556710

[B17] de Wied M. Verbaten M. N. (2001). Affective pictures processing, attention, and pain tolerance. Pain. 90, 163–172. 10.1016/S0304-3959(00)00400-0 11166983

[B18] Egle U. T. Egloff N. von Kanel R. (2016). [Stress-induced hyperalgesia (SIH) as a consequence of emotional deprivation and psychosocial traumatization in childhood: Implications for the treatment of chronic pain]. Berlin, Germany: Schmerz. 10.1007/s00482-016-0107-8 27324753

[B19] Egloff N. Camara R. J. von Kanel R. Klingler N. Marti E. Ferrari M. L.. (2014). Hypersensitivity and hyperalgesia in somatoform pain disorders. Gen. Hosp. Psychiatry. 36, 284–290. 10.1016/j.genhosppsych.2014.01.011 24650586

[B20] Engel G. L. (1959). Psychogenic pain and pain-prone patient. Am. J. Med. 26, 899–918. 10.1016/0002-9343(59)90212-8 13649716

[B21] Engel G. L. (1962). Guilt, pain, and success. Success facilitated by the pain of glomus tumor and peptic ulcer. Psychosom. Med. 24, 37–48. 10.1097/00006842-196201000-00007 13890218

[B22] Engel J. (2008). American Therapy: The Rise of Psychotherapy in the United States. London: Penguin Publishing Group.

[B23] Ercan I. Hafizoglu S. Ozkaya G. Kirli S. Yalcintas E. Akaya C.. (2015). Examinando los Puntajes de Corte para el Inventario de Ansiedad Estado-Rasgo. [Examining cut-off values for the State-Trait Anxiety Inventory.]. Revista Argentina de Clínica Psicológica. 24, 143–148.

[B24] Fishbain D. A. Cutler R. Rosomoff H. L. Rosomoff R. S. (1997). Chronic pain-associated depression: antecedent or consequence of chronic pain? a review. Clin. J. Pain. 13, 116–137. 10.1097/00002508-199706000-00006 9186019

[B25] Foa E. B. Cashman L. Jaycox L. Perry K. (1997). The validation of a self-report measure of posttraumatic stress disorder: the posttraumatic diagnostic scale. Psychol. Assess. 9, 445. 10.1037/1040-3590.9.4.445

[B26] Freud S. (1998). Das ökonomische Problem des Masochismus. GESAMMELTE WERKE: XIII: CHRONOLOGISCH GEORDNET, 371–83.

[B27] Giesbrecht T. Merckelbach H. Kater M. Sluis A. F. (2007). Why dissociation and schizotypy overlap: the joint influence of fantasy proneness, cognitive failures, and childhood trauma. J. Nerv. Ment. Dis. 195, 812–818. 10.1097/NMD.0b013e3181568137 18043521

[B28] Gilet A. L. (2008). [Mood induction procedures: a critical review]. L'Encephale. 34, 233–239. 10.1016/j.encep.2006.08.003 18558143

[B29] Godinho F. Frot M. Perchet C. Magnin M. Garcia-Larrea L. (2008). Pain influences hedonic assessment of visual inputs. Eur. J. Neurosci. 27, 2219–2228. 10.1111/j.1460-9568.2008.06196.x 18430033

[B30] Griesel D. Wessa M. Flor H. (2006). Psychometric qualities of the German version of the Posttraumatic Diagnostic Scale (PTDS). Psychol. Assess. 18, 262–268. 10.1037/1040-3590.18.3.262 16953729

[B31] Harder D. W. Greenwald D. F. (1999). Further validation of the shame and guilt scales of the Harder Personal Feelings Questionnaire-2. Psychol. Rep. 85, 271–281. 10.2466/pr0.1999.85.1.271 10575992

[B32] Hashmi J. A. Baliki M. N. Huang L. Baria A. T. Torbey S. Hermann K. M.. (2013). Shape shifting pain: chronification of back pain shifts brain representation from nociceptive to emotional circuits. Brain. 136, 2751–2768. 10.1093/brain/awt211 23983029 PMC3754458

[B33] Hutson S. P. Hall J. M. Pack F. L. (2015). Survivor guilt: analyzing the concept and its contexts. ANS Adv. Nurs. Sci. 38, 20–33. 10.1097/ANS.0000000000000058 25635503

[B34] Inbar Y. Pizarro D. A. Gilovich T. Ariely D. (2013). Moral masochism: on the connection between guilt and self-punishment. Emotion. 13, 14–18. 10.1037/a0029749 22985340

[B35] Jaeger B. C. Edwards L. J. Das K. Sen P. K. (2017). An R2 statistic for fixed effects in the generalized linear mixed model. J. Appl. Stat. 44, 1086–1105. 10.1080/02664763.2016.1193725

[B36] Kuhner C. Burger C. Keller F. Hautzinger M. (2007). [Reliability and validity of the Revised Beck Depression Inventory (BDI-II). Results from German samples]. Der Nervenarzt. 78, 651–656. 10.1007/s00115-006-2098-7 16832698

[B37] Lang P. J. Bradley M. M. Cuthbert B. N. (2005). International affective picture system (IAPS): digitized photographs, instruction manual unad affective ratings. Technical Report A-6. Gainesville, FL: University of Florida. 10.1037/t66667-000

[B38] Laux L. Glanzmann P. Schaffner P. Spielberger C. D. (1981). STAI; Das State-Trait-Angstinventar; Theoretische Grundlagen und Handanweisung. Beltz; Verlag, Psychologie.

[B39] Lewis H. B. (1971). Shame and guilt in neurosis. Psychoanal. Rev. 58, 419–438. 5150685

[B40] Lopez-Castro T. Saraiya T. Zumberg-Smith K. Dambreville N. (2019). Association between shame and posttraumatic stress disorder: a meta-analysis. J. Trauma. Stress. 32, 484–495. 10.1002/jts.22411 31291483 PMC7500058

[B41] Mills C. D'Mello S. (2014). On the validity of the autobiographical emotional memory task for emotion induction. PloS ONE. 9, e95837. 10.1371/journal.pone.0095837 24776697 PMC4002425

[B42] Mogil J. S. (2012). Sex differences in pain and pain inhibition: multiple explanations of a controversial phenomenon. Nat. Rev. Neurosci. 13, 859–866. 10.1038/nrn3360 23165262

[B43] Nakagawa S. Schielzeth H. A. (2013). general and simple method for obtaining R2 from generalized linear mixed-effects models. Methods Ecol. Evol. 4, 133–142. 10.1111/j.2041-210x.2012.00261.x30239975

[B44] Nelissen R. M. Zeelenberg M. (2009). When guilt evokes self-punishment: evidence for the existence of a Dobby Effect. Emotion. 9, 118–122. 10.1037/a0014540 19186924

[B45] Nelissen R. M. A. (2012). Guilt-induced self-punishment as a sign of remorse. Social Psychol. Personal. Sci. 3, 139–144. 10.1177/1948550611411520

[B46] Prager E. M. Chambers K. E. Plotkin J. L. McArthur D. L. Bandrowski A. E. Bansal N.. (2019). Improving transparency and scientific rigor in academic publishing. Brain Behav. 9, e01141. 10.1002/brb3.1141 30506879 PMC6346653

[B47] Regan D. T. Williams M. Sparling S. (1972). Voluntary expiation of guilt: a field experiment. J. Pers. Soc. Psychol. 24, 42. 10.1037/h0033553 5079553

[B48] Riley J. L. 3rd Robinson M. E. Wise E. A. Myers C. D. Fillingim R. B. (1998). Sex differences in the perception of noxious experimental stimuli: a meta-analysis. Pain. 74, 181–187. 10.1016/S0304-3959(97)00199-1 9520232

[B49] Riley J. L. 3rd Robinson M. E. Wise E. A. Price D. D. (1999). A meta-analytic review of pain perception across the menstrual cycle. Pain. 81, 225–235. 10.1016/S0304-3959(98)00258-9 10431710

[B50] Rolke R. Baron R. Maier C. Tolle T. R. Treede R. D. Beyer A.. (2006). Quantitative sensory testing in the German Research Network on Neuropathic Pain (DFNS): standardized protocol and reference values. Pain. 123, 231–243. 10.1016/j.pain.2006.01.041 16697110

[B51] Roy M. (2015). Cerebral and spinal modulation of pain by emotions and attention. in: Pain, Emotion and Cognition: A Complex Nexus, Eds Pickering G. Gibson S. (Cham: Springer International Publishing), 35–52. 10.1007/978-3-319-12033-1_3

[B52] Rusch N. Corrigan P. W. Bohus M. Jacob G. A. Brueck R. Lieb K.. (2007). Measuring shame and guilt by self-report questionnaires: a validation study. Psychiatry Res. 150, 313–325. 10.1016/j.psychres.2006.04.018 17320971

[B53] Schatzberg A. F. (2004). The relationship of chronic pain and depression. J. Clin. Psychiatry. 65(Suppl 12), 3–4. 15315470

[B54] Shi C. Ren Z. Zhao C. Zhang T. Chan S. H. (2021). Shame, guilt, and posttraumatic stress symptoms: a three-level meta-analysis. J. Anxiety Disord. 82, 102443. 10.1016/j.janxdis.2021.102443 34265540

[B55] Siqveland J. Hauff E. Ruud T. Brennen T. J. (2019). Posttraumatic stress and autobiographical memory in chronic pain patients. Scand. J. Pain. 19, 337–343. 10.1515/sjpain-2018-0044 30422805

[B56] Tangney J. P. Dearing R. Wagner P. E. (2000). The Test of Self-Conscious Affect – 3 (TOSCA-3). George Mason University, Fairfax VA. 10.1037/t06464-000

[B57] Tangney J. P. Stuewig J. Mashek D. J. (2007). Moral emotions and moral behavior. Annu. Rev. Psychol. 58, 345–372. 10.1146/annurev.psych.56.091103.070145 16953797 PMC3083636

[B58] Uhrig M. K. Trautmann N. Baumgärtner U. Treede R. D. Henrich F.. (2016). Emotion elicitation,: A., comparison of pictures and films. Front. Psychol. 7, 180. 10.3389/fpsyg.2016.00180 26925007 PMC4756121

[B59] Vachon-Presseau E. Centeno M. V. Ren W. Berger S. E. Tetreault P. Ghantous M.. (2016b). The emotional brain as a predictor and amplifier of chronic pain. J. Dent. Res. 95, 605–612. 10.1177/0022034516638027 26965423 PMC4924545

[B60] Vachon-Presseau E. Roy M. Martel M. O. Caron E. Marin M. F. Chen J.. (2013). The stress model of chronic pain: evidence from basal cortisol and hippocampal structure and function in humans. Brain. 136, 815–827. 10.1093/brain/aws371 23436504

[B61] Vachon-Presseau E. Tetreault P. Petre B. Huang L. Berger S. E. Torbey S.. (2016a). Corticolimbic anatomical characteristics predetermine risk for chronic pain. Brain. 139, 1958–1970. 10.1093/brain/aww100 27190016 PMC4939699

[B62] Wieser M. J. Pauli P. (2016). Chapter 1-Neuroscience of Pain and Emotion A2-al'Absi, Mustafa. in: Neuroscience of Pain, Stress, and Emotion, Eds Flaten M. A. (San Diego: Academic Press), 3–27. 10.1016/B978-0-12-800538-5.00001-7

[B63] Wilson J. P. Drozdek B. Turkovic S. (2006). Posttraumatic shame and guilt. Trauma, Violence Abuse. 7, 122–141. 10.1177/1524838005285914 16534148

[B64] Wingenfeld K. Spitzer C. Mensebach C. Grabe H. J. Hill A. Gast U.. (2010). [The German version of the Childhood Trauma Questionnaire (CTQ): preliminary psychometric properties]. Psychother. Psychosom. Med. Psychol. 60, 442–450. 10.1055/s-0030-1247564 20200804

[B65] Wrobel N. Wiech K. Forkmann K. Ritter C. Bingel U. (2014). Haloperidol blocks dorsal striatum activity but not analgesia in a placebo paradigm. Cortex. 57, 60–73. 10.1016/j.cortex.2014.02.023 24796219

[B66] Zhong C-B. Liljenquist K. (2006). Washing away your sins: threatened morality and physical cleansing. science. 313, 1451–1452. 10.1126/science.1130726 16960010

