# Supplementary material for: Guilt is effectively induced by a written auto-biographical essay but not reduced by experimental pain
Source: Front Behav Neurosci. 2022 Aug 11;16:891831. doi: 10.3389/fnbeh.2022.891831 (PMC9403731; doi:10.3389/fnbeh.2022.891831)
Supplement: Supplementary file 1 [file Data_Sheet_1.docx]

**Appendix 1: Guilt induction: Instruction for written auto-bio-essay appearing on screen.**

Important note to start with:

You are asked to write a text below, the content of which is very personal. Therefore, it will not be read by anyone and remains your private property. The text will always remain in your hands during and after the study. You alone decide whether, how, and when you want to destroy the record after completion of the study. This will ensure that no investigator, assistant, or other scientific staff involved in the study will be able to view or read your text.

Please describe a real-life event that truly happened, and on the occasion of which, you behaved very badly and unfairly towards a person closely acquainted with you. You disregarded important rules of conduct and your actions have hurt or may even done serious harm to the person who trusted you.

Please choose an event that is still unpleasant even now and that is still emotionally distressing for you. Perhaps, when remembering the event you also have the feeling that you have irretrievably destroyed something and that you could still be blamed for it. Or maybe you still blame yourself.

It is very important that you remember the event in question in as much detail and as vividly as possible. Please take your time before writing it down. Please also try to provide background information explaining how this injury occurred and what consequences your actions had for the person directly affected and for your relationship with her or him.

Please make every effort to not to leave out any detail and also to describe exactly how you feel and why you feel that way right now when remembering the incident.

Use the paper and pencil provided on the desk. You will be given 10 minutes. The computer will send a signal when time is up. If you finish earlier please read your text again and add any missing details.

**Guilt induction: Memory boost**

At the beginning of this experiment, you wrote a text. We ask you now to recall again exactly the event that really happened and in which you behaved badly and unfairly towards a close person.

Please try again to put yourself emotionally back into this unpleasant situation. In order to concentrate better, please close your eyes for 30 seconds. The computer will signal you when time is up.

**Appendix 2: Neutral induction: Instruction for written auto-bio-essay appearing on screen.**

Important note to start with:

You are asked to write a text below. The content of this text is not very personal. Nevertheless, it will not be read by anyone and remains your private property. The text will always remain in your hands during and after the study. You alone decide whether how and when you want to destroy the record after completion of the study. This will ensure that no investigator, assistant, or other scientific staff involved in the study will be able to view or read your text.

Please describe a real-life event that truly happened and where you met a person you know only fleetingly or not at all. The event should be an everyday encounter that took place without any significant mutual emotional involvement and was neither pleasant nor stressful for you.

Please choose an event that was emotionally neutral and is still neutral in your memory. Since you were not involved in either a positive or negative sense the encounter had no consequences and no deeper meaning for you. While remembering you may recall situations while shopping or on the tram.

Since it was a neutral encounter it is probably not possible for you to recall the event in great detail. Nevertheless, please take some time to write it down and try to record where and how this neutral meeting took place and how it was.

Since this was an everyday occurrence, and was of no emotional significance to you or the person you were meeting, details concerning feelings and emotions are not important. Therefore, you must only describe the situation itself and not how you feel about it right now.

Use the paper and pencil provided on the desk. You will be given 10 minutes. The computer will send a signal when time is up. If you finish earlier, please go through your text again and add any missing details.

**Neutral induction: Memory boost**

At the beginning of this experiment, you wrote a text. We ask you now to recall again exactly the event that really happened, and in which you met a person you know only fleetingly or not at all.

Please try again to put yourself back into this emotionally neutral situation. In order to concentrate better please close your eyes for 30 seconds. The computer will signal you when time is up.
